# The role of pannexin/purinergic signaling in intervascular communication from capillaries during skeletal muscle contraction in male Golden hamsters

**DOI:** 10.14814/phy2.16113

**Published:** 2024-06-19

**Authors:** Iain R. Lamb, Nicole M. Novielli‐Kuntz, Coral L. Murrant

**Affiliations:** ^1^ Department of Human Health and Nutritional Sciences University of Guelph Guelph Ontario Canada

**Keywords:** blood flow, capillary, muscle contraction, pannexin, skeletal muscle

## Abstract

We sought to determine the physiological relevance of pannexin/purinergic‐dependent signaling in mediating conducted vasodilation elicited by capillary stimulation through skeletal muscle contraction. Using hamster cremaster muscle and intravital microscopy we stimulated capillaries through local muscle contraction while observing the associated upstream arteriole. Capillaries were stimulated with muscle contraction at low and high contraction (6 and 60CPM) and stimulus frequencies (4 and 40 Hz) in the absence and presence of pannexin blocker mefloquine (MEF; 10^−5^ M), purinergic receptor antagonist suramin (SUR 10^−5^ M) and gap‐junction uncoupler halothane (HALO, 0.07%) applied between the capillary stimulation site and the upstream arteriolar observation site. Conducted vasodilations elicited at 6CPM were inhibited by HALO while vasodilations at 60CPM were inhibited by MEF and SUR. The conducted response elicited at 4 Hz was inhibited by MEF while the vasodilation at 40 Hz was unaffected by any blocker. Therefore, upstream vasodilations resulting from capillary stimulation via muscle contraction are dependent upon a pannexin/purinergic‐dependent pathway that appears to be stimulation parameter‐dependent. Our data highlight a physiological importance of the pannexin/purinergic pathway in facilitating communication between capillaries and upstream arteriolar microvasculature and, consequently, indicating that this pathway may play a crucial role in regulating blood flow in response to skeletal muscle contraction.

## 1 INTRODUCTION

The highly dynamic metabolic rate of skeletal muscle coupled with the fact that discrete motor units can be recruited within a larger muscle bed (Enoka, 1995) necessitates a level of vascular control that allows an increase in blood flow to be directed to individual working muscle fibers dispersed throughout a larger tissue during muscle contraction (Berg et al., 1997; Mackie & Terjung, 1983). Mounting evidence suggests that capillaries play an active role in coordinating this blood flow response within skeletal muscle during contraction (for review see Murrant et al., 2017). This is, in part, attributed to the fact that capillaries can respond to byproducts of muscle contraction to increase their own perfusion by initiating a vasodilatory signal transmitted to upstream arterioles controlling their perfusion (Berg et al., 1997; Cohen et al., 2000; Cohen & Sarelius, 2002; Lamb et al., 2018). The transmission of a vasodilatory signal, also known as a conducted or remote response, enables a coordinated vascular response along the length of the vessel by facilitating the movement of intervascular signal(s) between cells of the vascular wall; vascular smooth muscle and endothelial cells (Begandt et al., 2017; Figueroa & Duling, 2009). Currently, the primary signaling mechanism identified involves connexin‐ and gap junction (GJ)‐dependent spread of hyperpolarization (for review see Bagher & Segal, 2011). While there is evidence that contraction‐induced conducted responses initiated at the capillary may involve GJs, the hyperpolarization associated with GJ‐dependent communication has not been consistently observed (Cohen & Sarelius, 2002). Further, the characteristic bidirectional spread of hyperpolarization via GJs such as those induced by acetylcholine (ACh) (Budel et al., 2003; Duling & Berne, 1970) are in contrast with the directional, coordinated pathway of arteriolar vasodilation that is observed when capillaries are stimulated by contracting muscle fibers (Berg et al., 1997; Twynstra et al., 2012). Taken together, the distinct differences between the characteristics of GJ‐dependent communication and conducted responses induced by muscle contraction suggests the presence of a second signaling pathway that functions independently of hyperpolarization and possesses directional signaling capabilities.

We have recently demonstrated that capillaries can communicate with upstream arterioles through the involvement of pannexin (PanX) channels and purinergic membrane receptors (Pur) when stimulated with adenosine and potassium (Lamb et al., 2021), vasodilatory molecules shown to be present during muscle contraction (for review see Clifford & Hellsten, 1985). PanX channels have been implicated in mediating cellular signaling in various tissues (for review see Penuela et al., 2013). The PanX1 channel isoform has been found to be heavily expressed in both endothelial cells and vascular smooth muscle cells of the vasculature (Bao et al., 2004; Begandt et al., 2017; Lohman et al., 2012). PanX‐dependent signaling between cells functions independently of hyperpolarization and involves the cellular release of ATP through PanX channels and subsequent activation of Pur receptors on adjacent cells. Therefore, PanX and Pur mechanisms create a second pathway for communication along the microvascular wall that may contribute to coordinating blood flow during muscle contraction.

Given that we have previously demonstrated PanX/Pur‐dependent communication from capillaries to upstream arterioles in skeletal muscle, we sought to determine whether this pathway was physiologically relevant and could be stimulated by muscle contraction. To do this we stimulated capillaries through tetanic muscle contraction in the absence and presence of pharmacological antagonists targeting PanX/Pur‐ and GJ‐dependent pathways. Muscle stimulation parameters were chosen specifically guided by results of Dua et al., (2009) which determined that arteriolar vasodilation resulting from skeletal muscle contracting at high contraction frequencies was more dependent on potassium (K^+^) and adenosine (ADO), while low contraction frequencies were more dependent on nitric oxide (NO) and ADO, and arteriolar vasodilation resulting from contractions at high stimulus frequencies were more dependent on NO and low stimulus frequencies more dependant on NO and ADO. Therefore, we used skeletal muscle contraction induced by stimulation parameters that included high and low stimulus and contraction frequencies to stimulate the capillaries to determine whether different contraction parameters, therefore different vasodilators, resulted in differential upstream signaling through PanX/Pur and/or GJ‐dependent pathways.

## 2 METHODS

All experiments were approved by the Institutional Animal Care Committee Review Board at the University of Guelph and were conducted in accordance with the guidelines of the Canadian Council on Animal Care (CCAC). All animals had continuous access to food (Teklad irradiated rat chow T2918‐15, Inotiv, West Lafayette, IN) and water. Following all experimental protocols animals were euthanized with an overdose of sodium pentobarbital (0.26 mg/mL iv to effect).

### 2.1 General protocol: Preparing the cremaster muscle for experimentation

Adult male Golden Syrian hamsters (100–188 g) (*n* = 84) were anesthetized with sodium pentobarbital (70 mg/kg, i.p.), tracheotomized and catheterized using polyethylene catheters (outer tip diameter approx. 0.5 mm) placed in the left femoral vein for supplemental sodium pentobarbital infusion (10 mg/mL saline, 0.56 mL/h) throughout the experimental protocol. The animal was placed on an acrylic platform. Esophageal temperature was maintained at 37°C via convective heat from a coiled water‐filled glass tube (42°C) secured under the hamster. The right cremaster was prepared for in situ microscopy as described originally in Baez (1973) and modified by Murrant (2005). Briefly, a lateral longitudinal cut was made in the scrotum. The skin and fascia were separated from the cremaster muscle. The isolated cremaster was then cut longitudinally and separated from both the testis and epididymis. After separation, the testicle was pushed into the abdominal cavity. The cremaster muscle was spread over a semicircular Lucite plate. The edges of the cremaster were secured to the Lucite plate by insect pins to maintain muscle tension. Throughout the cremaster isolation surgery and all experimental protocols, the cremaster muscle was constantly superfused with a physiological salt solution (PSS) containing (in mmol/L) 131.9 NaCl, 4.7 KCl, 2.0 CaCl_2_, 1.2 MgSO_4_, 30 NaHCO_3_ and 0.3 mg/L tubocurarine hydrochloride pentahydrate. Tubocurarine chloride was added to the superfusate to block nicotinic, cholinergic membrane receptors to ensure muscle fibers were stimulated to contract directly using a microelectrode and not through the motor nerve. A physiological pH (7.35–7.45) of PSS was attained and maintained by aeration with 5%CO_2_ and 95%N_2_ gas. Cremaster muscle temperature was maintained by heating the superfusion solution to 42°C and adjusting the drip rate to achieve 34 ± 0.5°C. Following the cremaster isolation surgery, the hamster was transferred onto the microscope stage and allowed to equilibrate for 45‐60 min prior to data collection.

Visualization of the microvasculature of the cremaster muscle was achieved via transillumination with a tungsten lamp and an Olympus BX51WI microscope (Olympus Canada Inc., Richmond Hill, ON, Canada) using a ×20 long working distance water immersion objective (numerical aperture 0.40). The microscope image of the cremaster microvasculature was displayed on a video monitor using a video camera (DC220, Dage‐MTI, USA) and digitized using video compression software (EZ Grabber, Geniatech, China). Final magnification of the site was approx. ×2000. Diameter measurements were reproducible to within ±0.3 μm (*n* = 10).

**FIGURE 1:**
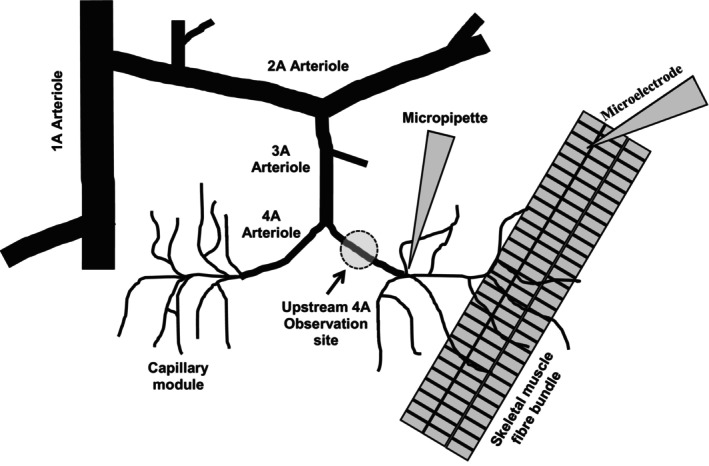
Schematic Representation of the Experimental Site. Schematic depicting part of the arteriolar microvascular network (feed arteriole, 1A; transverse arteriole, 2A; branch arteriole, 3A; module inflow arteriole, 4A) and associated capillaries. The diagram indicates the set‐up of different protocols that used either capillary stimulation by micropipette drug application or microelectrode stimulation and the modular inflow arteriolar observation site. Schematic not drawn to scale.

The specific architecture required for the experimental site was identified within the cremaster preparation. This experiment required the identification of a capillary module where at least one capillary remained in focus and could be traced back to its associated 4A arteriole upstream and this capillary had muscle fibers running underneath it that could be stimulated. Muscle fiber bundles underlying the capillary module were stimulated while the diameter of the upstream 4A arteriole diameter was monitored (Figure 1).

#### 2.1.1 Skeletal muscle contraction

Muscle fiber bundles (three to five fibers) underlying a capillary module (Figure 1) were stimulated directly using a silver wire microelectrode (tip diameter approx. 100 μm) placed in close proximity to, but not touching, muscle fibers running underneath the capillary module (Figure. 1). The microelectrode was positioned approximately 1000 μm away from the stimulated capillaries and at least 1000 μm away from the 4A arteriole observation site. The ground electrode was placed in the superfusate around the outer rim of the tissue support pedestal. Each stimulus was a square wave pulse of 0.4 ms duration and 8–15 V (Grass S48 stimulator, Quincy, MA, USA.). The voltage was adjusted to maximally stimulate four to five muscle fibers and then kept constant throughout the duration of the experiment.

Muscle contraction is produced through discrete bursts (trains) of multiple action potentials. Contractile activity of the muscle, and therefore the metabolism of the muscle, can be altered by changing four independent variables: (1) the action potential frequency within the train, or stimulus frequency within a train reported in Hz, (2) the duration of the train reported in milliseconds, (3) the number of trains delivered per minute or train or contraction frequency reported in contractions per minute (CPM), and (4) the total duration of the contraction period. During all experiments we kept the train duration constant at 250 ms and the total contraction time for each contraction bout was 2 min. We altered contraction frequency (6 or 60CPM) while keeping stimulus frequency constant (20 Hz) or altered stimulus frequency (4 or 40 Hz) while keeping contraction frequency constant (15CPM). A stimulus frequency of 4 Hz produced twitch contractions, contractions of 20 Hz stimulus frequency produced unfused tetanic contractions and contractions at 40 Hz produced fused tetanic contractions.

#### 2.1.2 Micropipette blocker application

Using a micromanipulator (Narishige, East Meadow, NY, USA) the tip of glass, drug‐filled micropipettes (tip diameter approx. 10 μm) were placed as close to the endothelial wall of a capillary as possible without touching the vessel or overlying tissue and positioned approximately 1000 μm from the upstream 4A arteriolar observation site between the capillary stimulation site and the 4A observation site. Pharmacological agents were ejected from the micropipette via pressure ejection through a water manometer (approx. 30cmH_2_O) and applied to a small region of the capillary (approx. 200 μm) as previously described in Frame & Sarelius (1995) and Murrant & Sarelius (2002). 100 μM fluorescein isothiocyanate‐dextran (FITC; FD‐150S; Sigma‐Aldrich, St. Louis, MO, USA) was added to the micropipette solution so brief epifluorescence could be used to verify that the pipette contents flowed away from the upstream 4A arteriolar observation site to avoid direct exposure. Previous control experiments have demonstrated FITC has no significant changes in vessel diameter during a 2 min application (Lamb et al., 2018). We micropipette‐applied either pannexin blocker mefloquine hydrochloride (MEF; 10^−5^ M; M2319 Sigma‐Aldrich, St. Louis, MO, USA), purinergic receptor antagonist suramin (SUR; 10^−5^ M; S2671 Sigma‐Aldrich, St. Louis, MO, USA) or GJ inhibitor halothane (HALO; 0.07%; B4388 Sigma‐Aldrich, St. Louis, MO, USA) in between the capillary stimulation site and the upstream 4A arteriolar observation site to block transmission of the signal without interfering with the stimulation of the capillaries or the manifestation of the vasodilation at the upstream site.

#### 2.1.3 Protocols

One arteriole per cremaster was used per protocol. Arteriolar diameter at the upstream 4A observation site was continuously recorded for 1 min prior to capillary stimulation using muscle contraction. Muscle was then contracted under the capillaries at either 6 and 60CPM or 4 and 40 Hz for 2 min while diameter of the 4A arteriole was continuously recorded. Muscle contraction was then stopped and 4A arteriolar diameter was continuously recorded for 2 min following the cessation of capillary stimulation.

One of three pharmacological blockers, either 10^−6^ M SUR, 10^−5^ M MEF or 0.07% HALO, were then micropipette applied to the vasculature in between the capillary stimulation site and the upstream 4A observation site for 30 min. Muscle contraction was then repeated while measuring diameter at the 4A upstream arteriolar site. Blocker application was then stopped and the effect of the blocker was allowed to wash out for 30 min before the muscle contraction protocol was repeated.

Following each experiment, maximal arteriolar diameters were recorded after 2 min superfusion with 10^−2^ M sodium nitroprusside (NO donor; Sigma‐Aldrich, St. Louis, MO, USA), considered to produce maximal vasodilation (Murrant et al., 2014).

### 2.2 Data analysis and statistics

All experiments were analyzed offline. For each protocol arteriolar diameter was measured every 10 s during the recording period. Still images from digitized recording were captured every 10 s (±1 s) using FrameShot software (EoF Productions, USA) and arteriolar diameters measured via ImageJ software (NIH, http://rsbweb.nih.gov/ij/). Control baseline diameters were defined as the diameter of the arteriole prior to capillary stimulation. Experimental baseline diameter was defined as arteriolar diameter in the presence of the signal transmission blocker, prior to capillary stimulation. Only one arteriole was observed per cremaster preparation and “*n*” indicates the number of arterioles observed.

Statistical analyses were performed using GraphPad Prism 10.2 (GraphPad software, San Diego, CA, USA). Data are reported as mean ± standard deviation (SD). Group means for baseline and maximal diameters were compared using Student's *t*‐test. Group means over time were compared with a two‐way repeated‐measures ANOVA. When the ANOVA identified significant differences, a Fisher's least significant difference (LSD) test was used post hoc to determine when the diameter changes were significantly different. Differences were considered significant when *p* < 0.05.

## 3 RESULTS

Tables 1, 2, 3, 4 report the resting diameters of the 4A arterioles under control and experimental conditions as well as the maximum diameter of the blood vessels under observation. For the majority of perturbations, signal transmission blockers did not affect resting diameter, as control and experimental baseline arteriole diameters were similar. There was one condition where SUR appeared to affect resting diameter, but this difference between baseline and experimental diameter was only relevant to the 6 CPM stimulation protocol dataset. Further, we found no significant differences in maximal diameter of the 4A arterioles under study. Lastly, none of the vascular stimulation conditions led to a maximal 4A vasodilation, indicating that the changes in recorded diameter were not restricted by reaching maximal diameter for the vessel.

**TABLE 1:** The average baseline and maximum arteriolar diameter for experiments testing the effect of pannexin inhibitor MEF on the conducted response elicited by muscle contraction. ‘*n*’ represents the number of 4A arterioles observed.

Figure	Protocol	Baseline diameter (μm)	Maximum diameter (μm)	*n*
2A	6CPM(20 Hz)	7.1 ± 3.1	15.3 ± 2.5	15
2A	6CPM(20 Hz) + MEF	7.0 ± 3.3		
3A	60CPM(20 Hz)	6.4 ± 2.4		
3A	60CPM(20 Hz) + MEF	7.4 ± 3.4		
4A	4 Hz(15CPM)	8.8 ± 3.3	19.4 ± 3.9 (*n* = 6*)	7
4A	4 Hz(15CPM) + MEF	8.7 ± 3.8		
5A	40 Hz(15CPM)	7.8 ± 2.2	18.6 ± 3.8	6
5A	40 Hz(15CPM) + MEF	7.1 ± 2.3		

*Note*: Values are mean ± SD.

* “*n*” values for maximal diameter differ from the “*n*” values for contraction data as the maximal diameter in one experiment was unable to be gathered.

**TABLE 2:** The average baseline and maximum arteriolar diameter for experiments testing the effect of purinergic membrane receptor inhibitor SUR on the conducted response elicited by muscle contraction. ‘*n*’ represents the number of 4A arterioles observed.

Figure	Protocol	Baseline diameter (μm)	Maximum diameter (μm)	*n*
2B	6CPM(20 Hz)	6.8 ± 2.0	14.6 ± 0.9	8
2B	6CPM(20 Hz) + SUR	5.4 ± 1.4*		
3B	60CPM(20 Hz)	5.8 ± 1.5		
3B	60CPM(20 Hz) + SUR	6.1 ± 1.2		
4B	4 Hz(15CPM)	9.0 ± 3.0	14.9 ± 2.5	13
4B	4 Hz(15CPM) + SUR	8.1 ± 3.0		
5B	40 Hz(15CPM)	7.5 ± 2.8	14.9 ± 2.4	12
5B	40 Hz(15CPM) + SUR	7.8 ± 2.3		

*Note*: Values are mean ± SD.

* Baseline Significantly differed from stimulation parameter without drug.

**TABLE 3:** The average baseline and maximum arteriolar diameter for experiments testing the effect of gap junction uncoupler HALO on the conducted response elicited by muscle contraction. ‘*n*’ represents the number of 4A arterioles observed.

Figure	Protocol	Baseline diameter (μm)	Maximum diameter (μm)	*n*
2C	6CPM(20 Hz)	6.9 ± 2.4	16.0 ± 2.9 (*n* = 8*)	9
2C	6CPM(20 Hz) + HALO	8.8 ± 3.5		
3C	60CPM(20 Hz)	7.6 ± 3.2		
3C	60CPM(20 Hz) + HALO	8.9 ± 4.7		
4C	4 Hz(15CPM)	8.1 ± 1.9	17.7 ± 4.3	9
4C	4 Hz(15CPM) + HALO	7.9 ± 2.1		
5C	40 Hz(15CPM)	7.0 ± 2.4		
5C	40 Hz(15CPM) + HALO	8.3 ± 2.0		

*Note*: Values are mean ± SD.

* “*n*” values for maximal diameter differ from the “*n*” values for contraction data as the maximal diameter in one experiment was unable to be gathered.

**TABLE 4:** The average baseline and maximum arteriolar diameter for experiments testing the effect of purinergic receptor inhibitors SUR and gap‐junction uncoupler HALO on the conducted response elicited by muscle contraction (protocol 7). ‘*n*’ represents the number of 4A arterioles observed.

Figure	Protocol	Baseline diameter (μm)	Maximum diameter (μm)	*n*
6	40 Hz(15CPM)	9.1 ± 3.5	18.1 ± 4.4	6
6	40 Hz(15CPM) + SUR+ HALO	10.4 ± 3.0		

*Note*: Values are mean ± SD.

### 3.1 Stimulation of capillaries via muscle contraction

**FIGURE 2:**
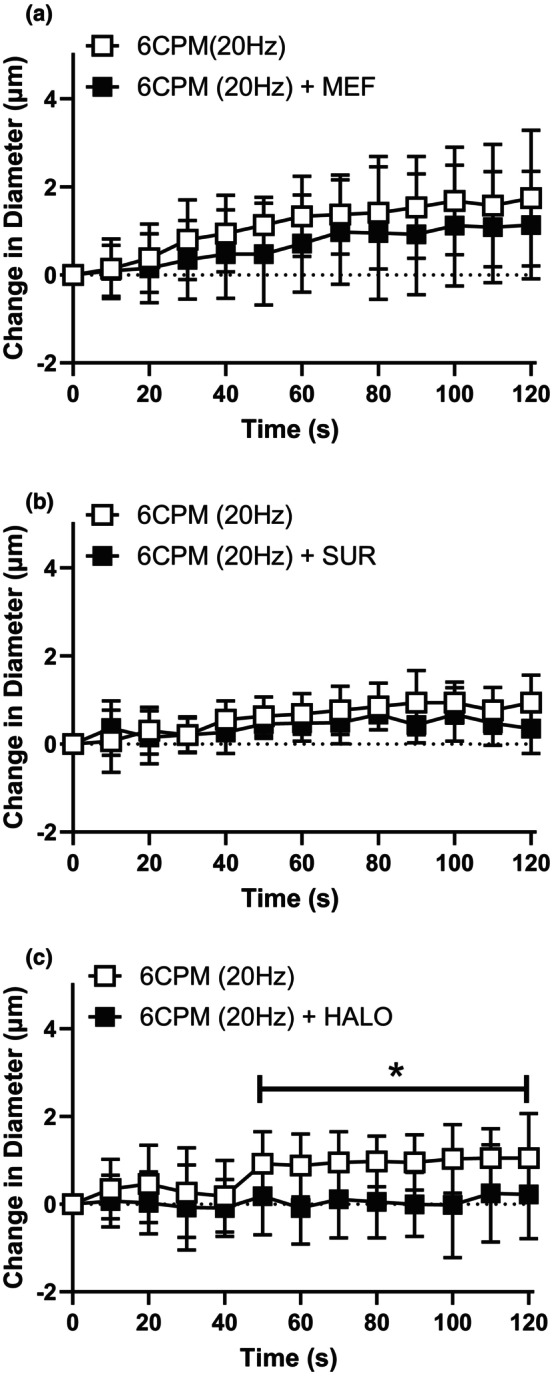
The conducted responses elicited by low contraction frequency was significantly blunted by the presence of gap‐junction uncoupler halothane. The change in diameter in of the 4A arteriole in response to capillary stimulation with low contraction frequency (6 CPM (20 Hz)) in the absence (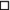) and presence (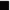) of (a) MEF (b) SUR and (c) HALO. Neither MEF treatment (*F* (1, 28) = 0.13; *p*_treatment_ = 0.72, *p*_time_ <0.0001, *p*_interaction_ = 1.0) nor SUR treatment (*F* (1, 14) = 1.89; *p*_treatment_ = 0.19, *p*_time_ <0.0001, *p*_interaction_ = 0.30) significantly impacted the vasodilation induced at 6CPM. HALO treatment did significantly inhibit the vasodilation induced by 6CPM muscle contraction (*F* (1, 16) = 4.8; *p*_treatment_ = 0.04, *p*_time_ = 0.0001, *p*_interaction_ = 0.034) and *indicates a significant difference between the contraction response in the absence and presence of the blocker. Data are shown as mean ± SD and analyzed by a two‐way repeated measures ANOVA with Fisher's LSD post hoc test where applicable. *F* statistics are reported with degrees of freedom for the numerator and the denominator (F [DFn, DFd]).

**FIGURE 3:**
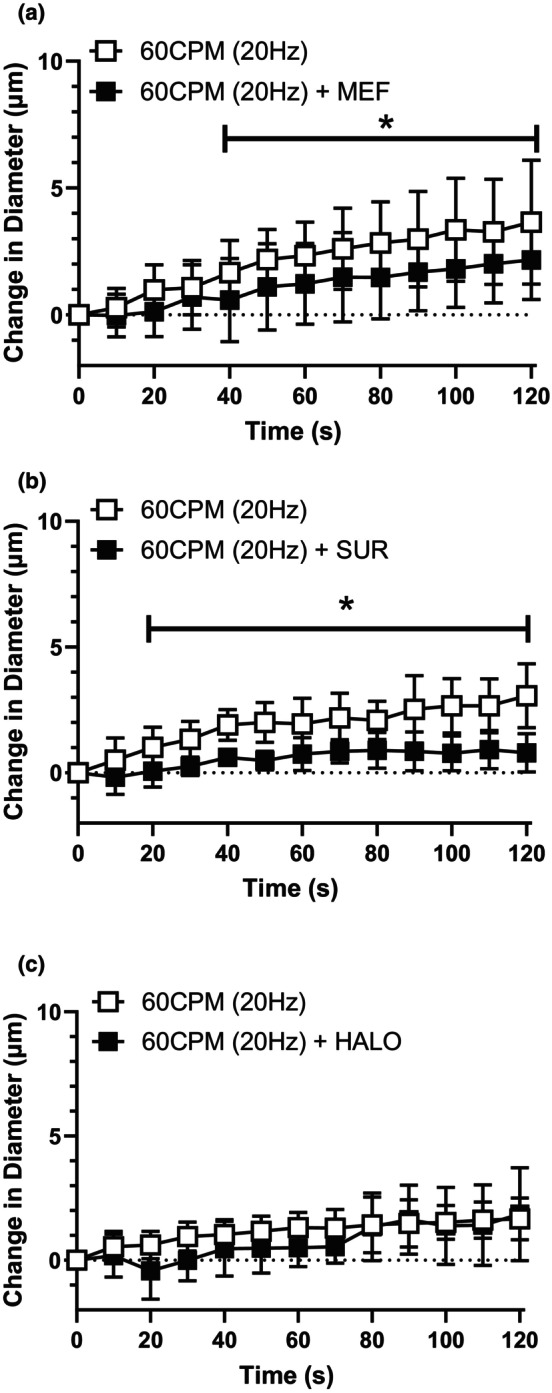
The conducted responses elicited by high contraction frequency was significantly blunted by the presence of pannexin inhibitor mefloquine and purinergic receptor blocker suramin. The change in diameter in of the 4A arteriole in response to capillary stimulation with high contraction frequency (60 CPM(20 Hz)) in the absence (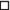) and presence (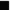) of (a) MEF (b) SUR and (c) HALO. Both MEF treatment (*F* (1, 28) = 5.4; *p*_treatment_ = 0.027, *p*_time_ <0.0001, *p*_interaction_ = 0.041) and SUR treatment (*F* (1, 14) = 27.5; *p*_treatment_ = 0.0001, *p*_time_ <0.0001, *p*_interaction_ = 0.0003) significantly impacted the vasodilation induced at 60CPM and *indicates a significant difference between the contraction response in the absence and presence of the blocker. HALO treatment did not significantly inhibit the vasodilation induced by 60CPM muscle contraction (*F* (1, 16) = 1.8; *p*_treatment_ = 0.20, *p*_time_ <0.0001, *p*_interaction_ = 0.17). Data are shown as mean ± SD and analyzed by a two‐way repeated measures ANOVA with Fisher's LSD post hoc test where applicable. *F* statistics are reported with degrees of freedom for the numerator and the denominator (F [DFn, DFd]).

Muscle contraction at a low contraction frequency (6 CPM) evoked a conducted vasodilation in the upstream 4A arteriole that was abolished in the presence of GJ uncoupler HALO (Figure 2c) but not significantly impacted by either the inhibition of PanX channels (via MEF, Figure 2a) or Pur receptors (via SUR, Figure 2b). Conversely, muscle contraction at a high contraction frequency (60 CPM) elicited an upstream vasodilation in the 4A arteriole that was significantly attenuated by both MEF (Figure 3a) and SUR (Figure 3b) but not HALO (Figure 3c). These findings suggest GJ‐dependent signaling has a more pronounced influence at lower contraction frequencies whereas the involvement of PanX/Pur‐dependent signaling is more prominent at higher contraction frequencies.

**FIGURE 4:**
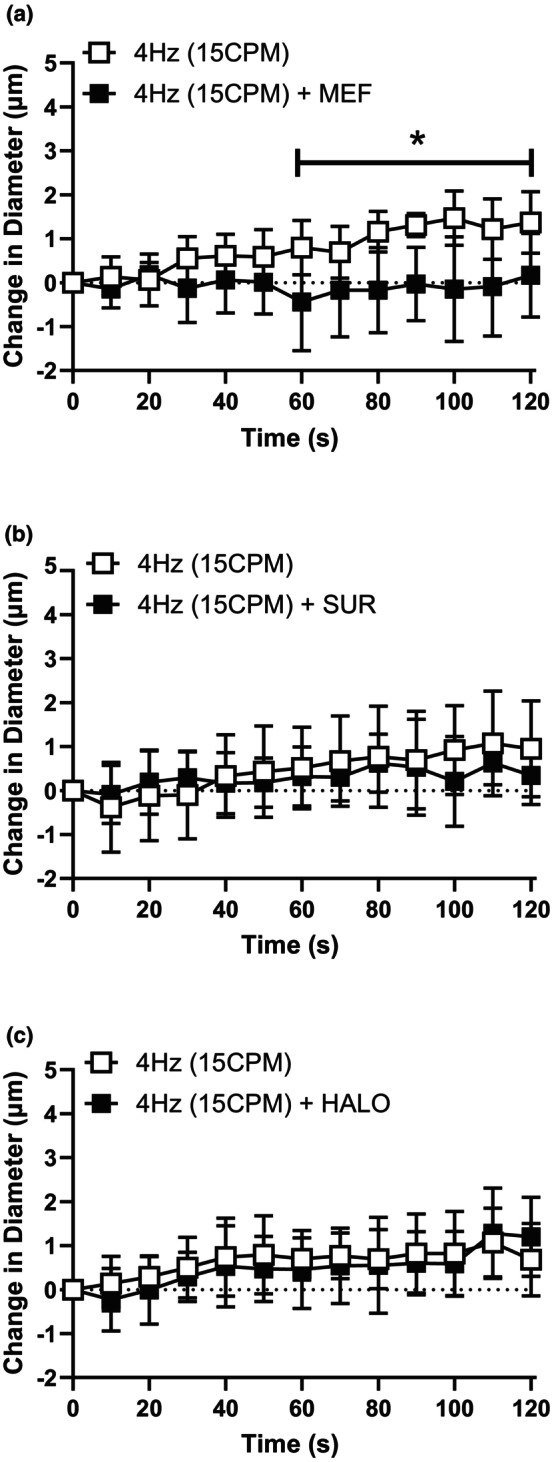
The conducted responses elicited by low stimulus frequency was significantly blunted by the presence of pannexin inhibitor mefloquine. The change in diameter of the 4A arteriole in response to capillary stimulation with low stimulus frequency (4 Hz [15CPM]) in the absence (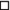) and presence (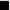) of (a) MEF (b) SUR and (c) HALO. MEF treatment (*F* (1, 12) = 9.4; *p*_treatment_ = 0.0097, *p*_time_ = 0.016, *p*_interaction_ < 0.0001) significantly impacted the vasodilation induced at 4 Hz and * indicates a significant difference between the contraction response in the absence and presence of the blocker. Both SUR treatment (*F* (1, 24) = 0.36; *p*_treatment_ = 0.56, *p*_time_ < 0.0001, *p*_interaction_ = 0.016) and HALO treatment did not significantly inhibit the vasodilation induced by 4 Hz muscle contraction (*F* (1, 16) = 0.27; *p*_treatment_ = 0.61, *p*_time_ < 0.0001, *p*_interaction_ = 0.53). *indicates a significant difference between the contraction response in the absence and presence of the blocker. Data are shown as mean ± SD and analyzed by a two‐way repeated measures ANOVA with Fisher's LSD post hoc test where applicable. *F* statistics are reported with degrees of freedom for the numerator and the denominator (F [DFn, DFd]).

**FIGURE 5:**
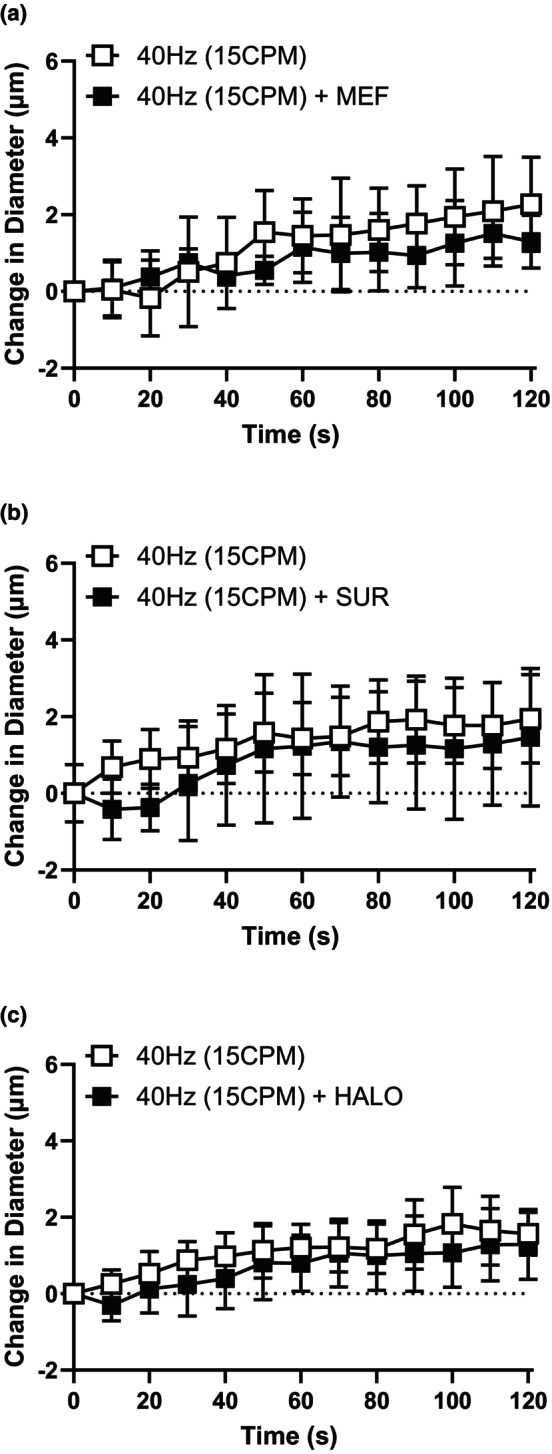
The conducted responses elicited by high contraction frequency was not significantly blunted by any of the tested antagonists. The change in diameter of the 4A arteriole in response to capillary stimulation with high stimulus frequency (40 Hz(15CPM)) in the absence (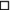) and presence (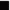) of (a) MEF (b) SUR and (c) HALO. Neither MEF treatment (*F* (1, 11) = 0.06; *p*_treatment_ = 0.82, *p*_time_ = 0.008, *p*_interaction_ = 0.98), SUR treatment (*F* (1, 23) = 1.73; *p*_treatment_ = 0.20, *p*_time_ < 0.0001, *p*_interaction_ = 0.076) or HALO treatment significantly impacted the vasodilation induced by 40 Hz muscle contraction (*F* (1, 16)=2.53; *p*_treatment_ = 0.13, *p*_time_ < 0.0001, *p*_interaction_ = 0.73). Data are shown as mean ± SD and analyzed by a two‐way repeated measures ANOVA with Fisher's LSD post hoc test where applicable. *F* statistics are reported with degrees of freedom for the numerator and the denominator (F [DFn, DFd]).

Muscle contraction at a low stimulus frequency (4 Hz) caused a conducted vasodilation in the associated upstream 4A arteriole that was significantly blunted in the presence of MEF (Figure 4a) but not effected by either SUR (Figure 4b) or HALO (Figure 4c), while the conducted vasodilation elicited by muscle contraction at high stimulus frequency (40 Hz) was not significantly attenuated by any of the antagonists tested (MEF, SUR or HALO, Figure 5). These observations indicate a possible role for PanX channels at lower stimulus frequencies but not at higher stimulus frequencies.

**FIGURE 6:**
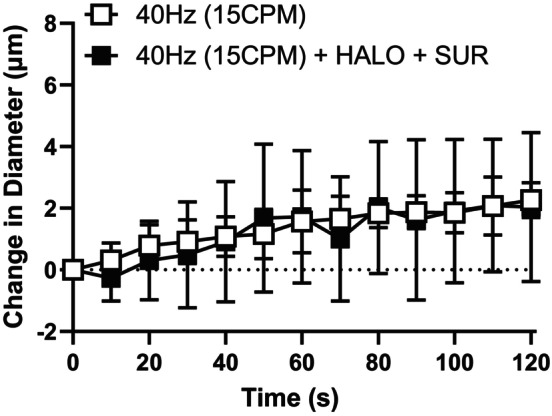
The conducted responses elicited by high stimulus frequency was not blocked by the combination purinergic receptor blocker suramin and gap junction uncoupler halothane. The change in diameter in of the 4A arteriole in response to capillary stimulation with high stimulus frequency (40 Hz (15CPM)) in the absence (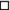) and presence (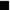) of SUR and HALO. The combination of HALO and SUR treatment (*F* (1, 10) = 0.036; *p*_treatment_ = 0.85, *p*_time_ < 0.0001, *p*_interaction_ = 0.85) did not significantly impact the vasodilation induced by 40 Hz muscle contraction. Data are shown as mean ± SD and analyzed by a two‐way repeated measures ANOVA with Fisher's LSD post hoc test where applicable. *F* statistics are reported with degrees of freedom for the numerator and the denominator (F [DFn, DFd]).

We further investigated the observation that conducted vasodilation elicited by 40 Hz muscle contraction was not inhibited by any individual blocker. It is possible, that with two transmission pathways present, that when one is blocked, the signal can switch to the alternate pathway. In order to determine whether the conducted response could switch from one pathway to the other we contracted muscle in the presence of blockers for both PUR receptors and GJ. In a separate set of experiments we contracted muscle for 2 min at 40 Hz in the absence and the presence of both HALO and SUR applied simultaneously (Figure 6). In the presence of the combination of the blockers, the conducted response produced by 40 Hz muscle contraction was not significantly blunted; however, there was a considerable increase in variability in the presence of both blockers indicating that the antagonists may be having a more complex, less uniform effect on the vasodilatory response under these conditions.

## 4 DISCUSSION

We show that muscle contraction stimulation of capillaries can induce upstream 4A arteriolar vasodilation through a PanX/Pur‐dependent pathway that can be used in conjunction with GJ‐dependent pathways, whereby the utilization of either the PanX/Pur‐ or GJ‐dependent pathway is dependant on the stimulation parameters used to contract the muscle (Table 5). These observations suggest that the metabolic activity of the muscle plays a role in determining how capillaries are stimulated and which conduction pathway transmits signals to the upstream arterioles. These data show that the conduction of signals along the vasculature through PanX/Pur mechanisms is a physiologically relevant pathway and underscores that capillaries are an active sensory component of the microvasculature that can detect and respond to changes in their local microenvironment.

**TABLE 5:** PanX/Pur and GJ signaling pathways contribute to contraction‐induced upstream conducted vasodilation from capillaries to arterioles. Results summarizing the affect (✓) or lack thereof (✗) of PanX/Pur or GJ antagonists on upstream arteriole conducted vasodilation elicited by various muscle contraction parameters at the capillary level.

Pathway	Drug	6CPM (20 Hz)	60CPM (20 Hz)	4 Hz (15CPM)	40 Hz (15CPM)
PanX/Pur	MEF	✗	✓	✓	✗
PanX/Pur	SUR	✗	✓	✗	✗
GJ	HALO	✓	✗	✗	✗
PanX/Pur + GJ	SUR + HALO	‐	‐	‐	✗

*Note*: ✗, Indicates the drug did not significantly affect the conducted response; ✓, Indicates the drug significantly affected the conducted response; ‐ Indicates drugs not tested.

### 4.1 PanX/Pur dependent responses

PanX channels have been implicated in mediating cellular signaling in various tissues (for review see Penuela et al., 2013). PanX‐dependent signaling between cells is proposed to involve the cellular release of ATP through PanX channels, the binding of ATP to P_2_ membrane receptors on an adjacent EC causing an increase in intracellular Ca^2+^ stimulating the opening of PanX channels causing the continued signaling of neighboring ECs (Begandt et al., 2017; Billaud et al., 2011; Good et al., 2015). Therefore, ATP release from skeletal muscle fibers during contraction (Hellsten et al., 1998; Lo et al., 2001; Mo & Ballard, 2001) could stimulate P_2_ membrane receptors on capillary endothelial cells and initiate intercellular communication through the PanX/Pur pathway. Red blood cells (RBCs) could also be a source of ATP during muscle contraction. RBCs can release ATP in response to reduced oxygen saturation, reduced pH and cellular deformation (Ellsworth et al., 2016; Kirby et al., 2021; Sprague et al., 1998), all of which may occur during muscle contraction. Both luminal and abluminal ATP could stimulate capillary endothelial cell P_2_ membrane receptors directly and result in the initiation of upstream signaling through the PanX/Pur pathway. ATP could also be a source of ADO which can, itself, stimulate capillaries and induce an upstream arteriolar vasodilation using the PanX/Pur pathway in skeletal muscle (Lamb et al., 2021). Extracellular ATP can be dephosphorylated to ADO by ecto‐nucleotide triphosphate diphosphohydrolase and ecto‐5′‐nucleotidases present on the extracellular membrane of skeletal muscle cells and vascular endothelial cells (Lynge et al., 2001; Yegutkin, 2008). ADO may stimulate the PanX/Pur pathway through receptor subtypes A_1_, A_2A_, and A_2B_ that have been identified on the vasculature in skeletal muscle (Lynge & Hellsten, 2000) with A_2A_ receptors on arterioles causing vasodilation via a stimulatory G‐protein‐coupled process involving increased cAMP and Ca^2+^ in downstream signaling processes (Maimon et al., 2014). The increase in intracellular Ca^2+^ may stimulate the opening of PanX channels and initiate the upstream response. Understanding the purinergic membrane receptor complement on capillary endothelial cells, determining whether ecto‐nucleotidases are on capillary endothelial cells in skeletal muscle and using ecto‐nucleotidases to accelerate ATP breakdown are all important next steps that would help with our understanding of whether ATP or ADO or both are responsible for the upregulation of the PanX/Pur pathway during contraction as well as provide information to suggest the cellular origin of the purinergic stimulus.

Potassium (K^+^) may also be involved in initiating an upstream response through a PanX/Pur‐dependent pathway during muscle contraction. We have identified K^+^ as a predominant vasodilator responsible for vasodilation at 60CPM (Dua et al., 2009) and K^+^, itself, stimulate capillaries and induce an upstream arteriolar vasodilation using the PanX/Pur pathway in skeletal muscle (Lamb et al., 2021). K^+^ may have a direct effect on opening PanX channels as elevated extracellular K^+^ as low as 10 mM has been shown to stimulate the opening of these channels (Suadicani et al., 2012). Additionally, in astrocytes, K^+^ has been shown to inhibit PanX channels inhibitors, thereby facilitating their opening (Jackson et al., 2014). Therefore, in our model, the release of K^+^ from contracting skeletal muscle could open PanX channels directly or inhibit inhibitors of the channels thereby facilitating the opening of PanX channels.

### 4.2 Contraction parameters and multiple conduction pathways

We used different contraction parameters as a tool to try to discern the contractile conditions in which the PanX/Pur signaling pathway, the GJ signaling pathway or both were involved in facilitating the conducted response from a capillary unit upstream to its 4A arteriole. Previously, we found that stimulating capillaries with either K^+^ or ADO directly induced a conducted response through a PanX/Pur‐dependent pathway while NO induced upstream signals using a GJ‐dependent pathway (Lamb et al., 2021). In a separate study using muscle contraction to stimulate 2A arterioles directly, Dua et al., (2009) found that the vasodilation from 4 Hz muscle contraction was primarily induced via NO and ADO, vasodilation resulting from 40 Hz contractions was primarily produced by NO, vasodilation resulting from muscle contracting at 6CPM was primarily produced through NO and ADO and vasodilation from 60CPM was primarily produced by ADO and K^+^. Based on these two studies, we anticipated that muscle contraction at 4 Hz or 6CPM would produce vasodilation primarily via NO and ADO and would have used both the GJ‐ and PanX/Pur‐dependent pathways. Additionally we hypothesized that 40 Hz muscle contraction would produce vasodilation via NO and would use the GJ‐dependent pathway and 60CPM muscle contraction would induce vasodilation via ADO and K^+^ and would use the PanX/Pur‐dependent pathway to signal arterioles upstream. For the most part, we did not observe these results. The only observation consistent with this logic was at 60CPM, where the conducted vasodilation was PanX/Pur‐dependent and not GJ‐dependent. Our inability to predict the specific pathways based on a “predominant” vasodilator identified under specific contractile conditions may stem from complex interactions and integrated signaling between “predominant” vasodilators and a host of other vasodilators present simultaneously as a result of muscle contraction (Clifford & Hellsten, 1985; Dua et al., 2009; Hong & Kim, 2017; Murrant & Sarelius, 2002).

Interestingly, irrespective of how muscle fibers were stimulated, our observations indicate that both pathways were not active at the same time. While we have previously observed that capillary stimulation with ACh produced upstream 4A vasodilation using both PanX/Pur‐ and GJ‐dependent pathways simultaneously (Lamb et al., 2021), it appears muscle contraction does not initiate both pathways simultaneously, even when using stimulation conditions that should have elicited upstream signaling using both pathways (4Hz and 6CPM). This suggests a specific pathway may be dominant under certain contractile (stimulation) conditions with the potential to change under different contractile (stimulation) conditions. It is also possible that both pathways are stimulated at the capillary level but integration of signaling pathways occur following stimulation, leading to the emergence of a predominant pathway to carry the signal.

The presence of multiple pathways for signaling the upstream microvasculature from the capillaries is not unexpected given the importance of these processes that direct blood flow to active skeletal muscle fibers during increased metabolic demand. The processes involved in active hyperemia have been shown to use multiple vasodilators, in a potentially redundant manner, to stimulate the vasculature. In a similar fashion, it stands to reason that there would be multiple, possibly redundant mechanisms that the vasculature would use to spread this signal to the upstream vasculature network to ensure that blood flow meets metabolic demand. We have demonstrated two pathways active during muscle contraction in the current study, but our 40 Hz data indicate there may be an additional pathway that has not yet been characterized. The inability to block the 40 Hz vasodilation in the presence or both PanX/Pur and GJ blockers applied at the same time indicates that the 4A vasodilation occurred via a third pathway coupling communication between capillaries and arterioles. There have been suggestions of different GJ‐dependent pathways with different conduction characteristics depending on the connexin compliment that comprises the GJ (de Wit, 2010; Figueroa & Duling, 2008) but the GJ connexin protein compliment in skeletal muscle capillaries is unknown. Therefore, continued investigation into other potential signaling mechanisms underlying conducted responses in capillaries of skeletal muscle is warranted.

During muscle stimulation and contraction associated with human movement there will be multiple frequencies stimulating muscle fibers to generate the force required for a given activity, and a variety of contraction frequencies to perform specific tasks, and each may change within one task. Therefore, further investigation using more complex muscle contraction patterns will help to better understand whether the pathways are stimulated by specific dilators independently, or whether discrete contraction conditions elicit the initiation of one signaling pathway over another. Finally, we will have to work to understand whether these pathways interact in a redundant manner and determine whether there are more pathways that exist to ensure this critical communication always occurs from muscle cells to capillaries to 4A arterioles.

### 4.3 Experimental considerations

Females were not included in the current study. The cremaster, a muscle unique to males, was used based on its suitability for intravital microscopy microvascular network studies particularly in conjunction with muscle contraction under capillaries. This model is an ideal testing ground for future work in the female microvasculature, where targeted experiments can be translated to a female model. We are currently investigating the use of the retractor preparation, present in both males and females, to determine if similar blood flow mechanisms exist in females.

While the cremaster is ideal for contracting muscle in relation to specific parts of the vasculature, it is important to consider that its muscle fibers are long and will stimulate other parts of the vasculature along its length when stimulated. Given the interconnectedness of the vascular network, it is plausible that conducted signals reaching the 4A observation site may originate from the upstream 2A arteriole rather than the stimulated capillary. We would expect that if conducted responses from other parts of the vasculature were influencing vasodilation of the 4A arteriole, the blockers placed between the capillary and the 4A arteriole would not be effective. Further, previous work has shown that light/dye ablation of endothelial cells between the capillaries and the upstream 4A arteriole abolishes the 4A vasodilation in response to stimulation of the capillary by muscle contraction, indicating that conducted responses observed in the 4A arteriole were predominantly the result of vasodilatory signals originating from downstream capillaries and not from other upstream vascular components (Lamb et al., 2018). Therefore, while we cannot definitively rule out the possibility of some influence from conducted vasodilations originating from other parts of the network, its influence does not contribute significantly to our observed 4A vasodilation.

In all conditions except one, the control baseline diameters for each contraction condition did not significantly differ from their corresponding experimental baseline. This implies the application of the blocker did not influence the diameter of arteriolar observation site. Similarities in diameter indicates that the smooth muscle around each vessel had a similar length at the beginning of the stimulus. As force developed in smooth muscle (contraction or dilation) is dependent on length (Davis & Gore, 1989; Seow, 1985), we can be confident that the vasodilation induced by the stimulus was not affected by smooth muscle length and changes in the ability of the smooth muscle to generate force. The single instance where there was a significant difference between control and experimental baseline for 6CPM contraction condition and with the drug SUR, the diameter difference was small enough to have little impact on force production considering the shape of the length‐tension curve in smooth muscle (Davis & Gore, 1989; Seow, 1985).

Pericytes are found at the capillary level in skeletal muscle, yet their function remains poorly understood (for review see Murray et al., 2017). The population of pericytes associated with the skeletal muscle vasculature is not well defined, where the coverage has been estimated as low as 1:100 pericyte to endothelial cells (Diaz‐Flores et al., 2009; Shepro & Morel, 1993) to extensive pericyte coverage of skeletal muscle capillaries reported in a more recent study (Attrill et al., 2020). If pericytes form a continuous layer on capillaries and share the same capillary unit structure, there is the possibility they may play a role in signal transmission along the vasculature. While studies have shown the communication between capillaries and their 4A arterioles are dependent on endothelial cell integrity (Lamb et al., 2018) understanding if there is a connected pericyte network and its network structure will help contribute to determining their potential role in intervascular communication.

### 4.4 Summary

Our findings demonstrate that muscle contraction under capillaries can stimulate multiple vasodilatory pathways for capillaries to transmit conducted responses to upstream arteriolar elements; one pathway is reliant on GJ's, while the second is dependent upon PanX/Pur signaling. Further, our data suggest the presence of a third pathway that may contribute to the transmission of the conducted response initiated by capillary stimulation. Finally, our data support that capillaries serve as important metabolic sensors of the skeletal muscle microenvironment. When stimulated by changes in skeletal muscle metabolism, capillaries can transmit signals, in multiple ways, to their associated upstream 4A arterioles, leading to vasodilation and an increase in their own perfusion. These mechanisms position capillaries as central to the control of blood flow in contracting skeletal muscle.

## AUTHOR CONTRIBUTIONS

IL was responsible for conception and design of the experiments, data collection, analysis and interpretation of the data and writing of the manuscript. NN was responsible for data collection, analysis and interpretation of the data. CM was responsible for conception and experimental design, data analysis, data interpretation and writing the manuscript. All authors approved of the final version of the manuscript and agree to be accountable for all aspects of the work in ensuring that questions related to the accuracy or integrity of any part of the work are appropriately investigated and resolved. All persons designated as authors qualify for authorship, and all those who qualify for authorship are listed.

## FUNDING INFORMATION

This work was funded by Natural Sciences and Engineering Research Council of Canada, RGPIN‐2014‐05184.

## CONFLICT OF INTEREST STATEMENT

The authors have no conflict of interest to declare.

## ETHICS STATEMENT

All experiments were approved by the Institutional Animal Care Committee Review Board at the University of Guelph (Anumal Use Protocol Approval number #4562) and were conducted in accordance with the guidelines of the Canadian Council on Animal Care (CCAC).

## Data Availability

Data available on request.
